# Advanced sequencing approaches detected insertions of viral and human origin in the viral genome of chronic hepatitis E virus patients

**DOI:** 10.1038/s41598-022-05706-w

**Published:** 2022-02-02

**Authors:** C.-Patrick Papp, Paula Biedermann, Dominik Harms, Bo Wang, Marianne Kebelmann, Mira Choi, Johannes Helmuth, Victor M. Corman, Andrea Thürmer, Britta Altmann, Patrycja Klink, Jörg Hofmann, C.-Thomas Bock

**Affiliations:** 1 Division of Viral Gastroenteritis and Hepatitis Pathogens and Enteroviruses, Department of Infectious Diseases, Robert Koch Institute, Berlin, Germany; 2 Institute of Virology, Charité-Universitätsmedizin Berlin, Corporate Member of Freie Universität Berlin, Humboldt-Universität Zu Berlin, Berlin Institute of Health, German Centre for Infection Research, Berlin, Germany; 3 College of Veterinary Medicine, Virginia Polytechnic Institute and State University, Blacksburg, VA USA; 4 Department of Nephrology and Intensive Medical Care, Charité Universitätsmedizin Berlin, Corporate Member of Freie Universität Berlin and Humboldt-Universität Zu Berlin, Berlin, Germany; 5 Charité-Vivantes GmbH, Labor Berlin, Berlin, Germany; 6 Genome Sequencing, Methodology and Research Infrastructure, Robert Koch Institute, Berlin, Germany; 7 Institute of Tropical Medicine, University of Tübingen, Tubingen, Germany

**Keywords:** Virus structures, Viral evolution, Viral hepatitis

## Abstract

The awareness of hepatitis E virus (HEV) increased significantly in the last decade due to its unexpectedly high prevalence in high-income countries. There, infections with HEV-genotype 3 (HEV-3) are predominant which can progress to chronicity in immunocompromised individuals. Persistent infection and antiviral therapy can select HEV-3 variants; however, the spectrum and occurrence of HEV-3 variants is underreported. To gain in-depth insights into the viral population and to perform detailed characterization of viral genomes, we used a new approach combining long-range PCR with next-generation and third-generation sequencing which allowed near full-length sequencing of HEV-3 genomes. Furthermore, we developed a targeted ultra-deep sequencing approach to assess the dynamics of clinically relevant mutations in the RdRp-region and to detect insertions in the HVR-domain in the HEV genomes. Using this new approach, we not only identified several insertions of human (AHNAK, RPL18) and viral origin (RdRp-derived) in the HVR-region isolated from an exemplary sample but detected a variant containing two different insertions simultaneously (AHNAK- and RdRp-derived). This finding is the first HEV-variant recognized as such showing various insertions in the HVR-domain. Thus, this molecular approach will add incrementally to our current knowledge of the HEV-genome organization and pathogenesis in chronic hepatitis E.

## Introduction

Hepatitis E virus (HEV) is one of the main causes of acute viral hepatitis worldwide. It is a hepatotropic, approximately 7.2 kb single-stranded positive-sense RNA virus whose genome contains three open reading frames (ORF1, ORF2, and ORF3), 5′ and 3′ untranslated regions (UTRs), and a poly(A) tract at the 3′ end. The genotypes HEV-1 to HEV-8 belong to the genus *Orthohepevirus A* of which at least HEV-1 to HEV-4 can infect humans. HEV-1 and HEV-2 are responsible for large waterborne outbreaks in low-income countries whereas HEV-3 and HEV-4 cause foodborne infections and are autochthonous in high-income countries^1,2^. The latter two are zoonotic strains with mainly pigs and wild boars as putative main hosts and are transmitted to humans by the ingestion of raw or undercooked meat^3–5^. It has been shown that HEV-3 has a higher variability compared to other HEV genotypes and has been correlated with a higher host range^6^. Recombination events in this genotype have been repeatedly described, most frequently as insertions in the hypervariable region (HVR, also denoted as polyproline region, PPR)^7–11^. The inserted sequences mostly originated in HEV, however, sequences originating in the human genome have also been identified^7,12–14^. Recombinants and other HEV variants were associated with ribavirin treatment failure, adaptability in cell culture, and increased viral replication potential^10,12,13,15,16^. Besides recombination events, some single-nucleotide polymorphisms (SNP) such as G1634R were linked with RBV treatment failure^17^. Further SNPs identified in the RNA-dependent-RNA-polymerase (RdRp) coding sequence in patients treated with RBV were Y1320H, K1383N, K1398R, V1479I, Y1587F, G1634K^18^. Similar to other RNA viruses, HEV builds a so-called mutant cloud, which represents an intra-host heterogeneous population, with the advantage of rapid adaptation to environmental conditions^19^. Population-based Sanger sequencing has been shown to miss minor variants with a frequency below 20%^20^. Therefore, to capture the heterogeneity of the HEV quasispecies, including recombination events and SNP that occur with very low frequencies, we developed new sequencing approaches for HEV genotype 3 based on the amplification of the near full-length genome of HEV by long-range PCR (lrPCR) followed by subsequent next-generation sequencing (NGS) and third-generation sequencing. These methods allow the identification of insertions and SNPs in the HEV genome. Furthermore, single-molecule sequencing using Oxford Nanopore Technologies (ONT) enables the analysis of potential correlation in the occurrence of these events, as mutations and insertions can be detected on either the same or different DNA strands. A multiplex third-generation sequencing protocol (Oxford Nanopore Technologies, 1D^2^ flow cell) was also established as an additional optimization step to perform parallel sequencing of multiple samples. Amplicon-based NGS was performed to detect multiple insertions that coexist in the viral population and to determine the dynamic of SNPs in the RdRp region of the HEV genome.

## Material and methods

### Chronic hepatitis E patients and sample collection

To evaluate the newly established molecular methods, a total of nine plasma samples and one faecal sample from patients with chronic hepatitis E infection (CHE), defined as persistence of HEV RNA for longer than three months^21^, were used in this study. All patients were immunosuppressed (Table 1). Five samples were follow-up samples from one patient (Table 1). The samples 17-0421 and 18-0056 were used to compare the sequencing methods and for the sake of clarity will be referred to as *sample 1* and *sample 2,* respectively. All samples were obtained from routine diagnostics and were pseudonymised before usage. The HEV genotype for all samples was determined as described previously^22^.

**Table 1:** Samples from patients with chronic HEV infection.

Patient	Sample no	Sample name	Sample type	Cause of immunodeficiency	Collection date	Virus Concentration (copies/ml)
1	1a	17-0421–*sample 1*	Plasma	Atypical hemolytic-uremic syndrome, severe immunoglobulinaemia	10/2017	4.1 × 10^8^
1	1b	17–0420	Stool		10/2017	1.17 × 10^9^
1	2	17–0534	Plasma		11/2017	2 × 10^5^
1	3	18–0002	Plasma		01/2018	6.5 × 10^7^
1	4	18-0056–*sample 2*	Plasma		04/2018	8.5 × 10^7^
2	5	17–0371	Plasma	Kidney and pancreas transplantation	07/2017	4.4 × 10^6^
3	6	17–0535	Plasma	Kidney transplantation	12/2017	1.6 × 10^5^
4	7	18–0058	Plasma	Selective IgG deficiency, T-lymphopenia with helper cells < 200	04/2018	1.3 × 10^6^
5	8	18–0066	Plasma	Peripheric T-cell lymphoma	01/2018	1.2 × 10^6^
6	9	18–0068	Plasma	Kidney transplantation	04/2018	8.1 × 10^6^

### RNA extraction, cDNA synthesis, and long-range PCR (lrPCR)

RNA extraction was performed using QIAcube (QIAGEN, Hilden Germany) with the QIAamp Viral RNA Mini kit (QIAGEN, Hilden, Germany) according to the manufacturer's instruction. The RNA obtained from each sample was stored at − 80 °C until use. Quantitative PCRs for HEV were performed as previously described^22,23^. Isolated RNA was used for cDNA synthesis with the SuperScript IV First-Strand Synthesis System (Invitrogen, Thermo Fisher Scientific, Carlsbad, CA, USA). The synthesis was performed with Oligo d(T) primers and 11 µL template RNA as described in the manufacturer’s protocol with minor modification of the cDNA synthesis step at 60 °C for 20 min. To verify the successful synthesis of the whole HEV genome, four genome-wide PCRs were performed. Subsequently, the cDNA was used for the near full-length lrPCR. The lrPCR was performed using the Kapa HiFi Readymix (Sigma-Aldrich, Merck KGaA, Darmstadt, Germany) with an optimised program for GC-rich templates for both, first and heminested PCR. Primers and PCR protocols are listed in the Supplementary Tables S1 and S2. The PCR products were visualized on a 1% agarose gel using the BioDocAnalyze software (Biometra GmbH, Göttingen, Germany).

### Illumina sequencing of the lrPCR products and reads processing

Magnetic beads purification of the lrPCR products was performed with the MagSi-NGS Prep Plus kit (magtivio B.V., The Netherlands) according to the manufacturer’s instructions. The purified PCR products were measured by Qubit fluorometer using the double-stranded DNA High Sensitivity Assay Kit (Thermo Fisher Scientific, Carlsbad, CA, USA). A whole-genome (WG) library was prepared for all PCR products using the Nextera XT library kit (Illumina, San Diego), following the manufacturer`s instructions. WG libraries were sequenced in a 2 × 250 bp sequencing run on an Illumina HiSeq 2500 platform (Illumina Inc., San Diego, USA).

Illumina raw sequencing data were converted using bcl2fastq v 1.8.4 conversion software (Illumina Inc., San Diego, USA) and demultiplexed according to their multiplex identifier. For each sample, two fastq files were generated representing the paired-end reads. These two files were imported into Geneious version 11.1.5 (Biomatters Ltd, Auckland, NZ) and automatically paired. Adapter and quality trimming was performed using BBDuk Adapter/Quality Trimming Version 37.64 by Brian Bushnell implemented in Geneious. Minimum quality was set to 30. Trimmed reads were mapped to the reference sequence wbGER27_RAS (FJ705359.1) using Geneious mapper with medium sensitivity and performing five iterations. The consensus sequence containing the most common bases was extracted.

### Oxford Nanopore sequencing of the lrPCR products and reads processing

Third-generation sequencing for long-read sequencing was performed using Oxford Nanopore Technologies (ONT) MinION. To multiplex samples on the high accuracy 1D^2^ flow cells (Oxford Nanopore Technologies, Oxford, UK), the manufacturer’s native barcoding protocol (XP-NBD104) recommended for Ligation-Libraries (SQK-LSK109) was combined with the Ligation 1D^2^ (SQK-LSK308) manufacturer’s protocol. Barcoding of all samples was performed according to the ONT 1D Native barcoding genomic DNA protocol. After barcoding, dA-tailing of the barcoded DNA was performed followed by magnetic beads clean-up and the ONT 1D^2^ adapter ligation step from the 1D^2^ sequencing of the genomic DNA protocol. After 1D^2^ adapter ligation, elution and DNA concentration measurements using a Qubit fluorometer (Thermo Fisher Scientific) were performed for each sample. The prepared samples were pooled to a final mix with a volume of 50 µL and a concentration of approximately 10 ng/µL. The prepared sample pool was used for sequencing according to the 1D^2^ sequencing protocol.

The raw reads were processed and demultiplexed using ONT Guppy basecaller (Oxford Nanopore Technologies, Oxford, UK). Post-analysis of the resulting fastq files was done with Geneious version 11.1.5 (Biomatters Ltd, Auckland, NZ). Due to the high amount of data generated by this sequencing approach, the lack of NGS data for ONT read correction for samples 6–10, and the limited computational power available, only the reads for *sample 1* and *2* were analysed. Therefore, sequences with lengths between 6.5 and 8 kb were extracted. In the size selected sequence list, each insertion found was annotated. The function “extract annotation” was used to extract the entire sequences based on their annotation. To validate the ONT reads that contain two insertions simultaneously, the trimmed and error-corrected Illumina reads were mapped against the MinION sequence from *sample 2* which contained two insertions simultaneously. Mapping was performed using Geneious mapper at medium sensitivity, executing five iterations. The consensus sequence containing the most common bases was extracted. In addition, the size selected long-reads from *sample 2* were mapped against a reference (GenBank ID: FJ705359.1) using Geneious mapper set at medium sensitivity. The consensus sequence containing the most common bases was extracted.

### Amplicon-based NGS

Target specific primers containing flow cell adapters at the 5’ end were designed (Supplementary Table S2). These primers were used to generate amplicons containing the region of interest which were subsequently sequenced on Illumina MiSeq using the MiSeq Reagent Kit v3 (Illumina, San Diego, USA) generating 2 × 300 bp reads. The amplicons are between 350 and 500 bp in size allowing two paired reads to overlap. Merging the two reads of a pair results in a single end-to-end-sequence covering potential insertions with high accuracy.

RNA was isolated as described above and used for cDNA synthesis with the SuperScript IV First-Strand Synthesis System (Invitrogen, Thermo Fisher Scientific, Carlsbad, CA, USA) using random hexamers and 11 µL template RNA as described in the manufacturer’s protocol. In contrast to the cDNA synthesis performed for lrPCR, for the amplicon NGS, we used random hexamers increasing the cDNA yield compared to Oligo d(T) primers^24^.

### Hypervariable region amplicons

For the HVR amplicons, a fragment of approximately 800 bp in size was amplified. The resulting PCR products were used for nested PCR with primers containing overhang adapters for the Illumina flow cell (Supplementary Table S2). Depending on the length of insertion, the size of the generated amplicons varies. Therefore, two sets of primers aiming to generate the optimal amplicon size for NGS were designed. For amplification in the first and nested PCRs the Kapa HiFi Readymix (Sigma-Aldrich, Merck KGaA, Darmstadt, Germany) was used. The success of amplification and the amplicon size was verified by 1.5% agarose gel electrophoresis using the BioDocAnalyze software (Biometra GmbH, Göttingen, Germany).

### RNA-dependent RNA-polymerase amplicons

A 2 kb target sequence of the complete HEV polymerase region was amplified by TaKaRa Ex Taq DNA Polymerase (Takara Bio Inc., Japan) according to the manufacturer’s instructions using the primers listed in the Supplementary Table S2 with the PCR conditions described in the Supplementary Table S1. The PCR product was used as template for three approximately 550 bp overlapping PCR amplicons that cover the complete coding RdRp domain. Target specific primers with Illumina MiSeq overhang adapters were designed. Amplification was performed using the Takara Ex Taq DNA polymerase (Takara Bio Inc., Japan) with the PCR conditions described in the Supplementary Table S1. The success of amplification was verified by 1.5% agarose gel electrophoresis using the BioDocAnalyze software (Biometra GmbH, Göttingen, Germany).

### Amplicon preparation, sequencing, and reads processing

Magnetic beads purification of the PCR products was performed with the MagSi-NGS Prep Plus kit (magtivio B.V., The Netherlands) according to the manufacturer’s instructions. Purified PCR products were measured by Qubit fluorometer using the double-stranded DNA High Sensitivity Assay kit (Thermo Fisher Scientific). To compare the quality between a pooled and a separate sequencing approach, for sample 1b two different pools were prepared: one containing only the three RdRp amplicons, the second containing the RdRp and the HVR amplicons. These two pools were sequenced simultaneously using the Illumina MiSeq technology.

Amplicon libraries were generated using the Nextera XT library kit (Illumina, San Diego). The index-PCR was performed according to the manufacturer’s protocol. Indexed libraries were pooled and sequenced on the Illumina MiSeq using 2 × 300 bp reads. The read files containing paired-end reads provided for each sample were paired, trimmed, and mapped as described above.

### Single-nucleotide polymorphisms detection

Single-nucleotide polymorphisms (SNP) were detected using the Geneious “Find Variations/SNPs” tool. The minimum variant frequency was set to 0.005, maximum variant *P*-value to 10^–6^ and minimum strand-bias *P*-value at 10^–5^ when exceeding 65% bias. Regions below a coverage of 500 were excluded from variant calling.

### Cut-off for biological variants

cDNA synthesis, PCR, and the sequencing process are sources of error that need to be considered when sequencing RNA viruses. Therefore, we used the SuperScript IV high-fidelity RT which has a misincorporation frequency of 1.8 × 10^–4^^25^. *Sample 1* and *2* had high viral loads of 10^8^ and 10^6^ copies/ml, respectively (Table 1), which translates into a higher yield for the cDNA synthesis. Furthermore, to maximise the amount of cDNA synthesised and consequently used for lrPCR, a maximum of 11 µL template RNA was used for cDNA synthesis. The cut-off for biological variants was set at 0.5%.

Regarding sequencing errors, by trimming all bases with a Phred quality score lower than 30, the expected error rate is five times lower than our cut-off. The plausibility of the minority mutations detected was assessed by comparing their values with the values detected when other sequencing approaches were used or with the values detected in other follow-up samples.

### Calculation of polymorphisms and type of selection

In order to calculate the proportions of the polymorphism, the number of SNPs over 0.5% detected in a specific region was divided by the number of nucleotides in that region. To determine the type of selection in a specific region of the HEV genome, we used the ratio between nonsynonymous (dN) and synonymous (dS) substitutions per site (ratio dN/dS). Insertions in the HVR were excluded from the calculation.

### Detection of insertions

Due to the high variability of the HVR and to the targeted manner of the sequencing approach, the UNOISE3-Suite was used to get zero-radius operational taxonomic units (zOTUs) from the HVR-amplicon reads^26^. Thus, clustering of highly similar reads and generation of representative consensus sequences as zOTUs was performed. zOTU sequences were imported into Geneious and mapped to the reference sequence wbGER27_RAS (FJ705359.1). The origin of the HVR insertions was determined using the BLASTn search engine (https://blast.ncbi.nlm.nih.gov).

### Correlation between insertions and mutations

The long-read ONT sequences from *sample 1* and *2* were grouped based on their insertions. Accordingly, there were the *AHNAK insertion*, the *HEV-derived insertion,* and the *no insertion* sequence groups. The frequencies of the RdRp mutations G1634R, Y1587F, V1479I, and K1383N that were detected in the analysed samples were determined for each group and compared. A correlation between a certain mutation and an insertion would result in a higher frequency of this mutation in one of the groups. However, given a Phred score of 10 for our ONT reads, the frequencies of the mutations were interpreted as estimates.

### Accession numbers

HEV sequences described in this article have been submitted to NCBI GenBank under the accession numbers: MW837243 to MW837255 (Supplementary Table S3).

### Ethical approval

The ethics committee of the Charité Universitätsmedizin Berlin approved the study (approval number No. EA1/367/16) and written informed consent was obtained from all participating individuals. Patient samples were de-identified for this study. All experiments were performed in accordance with relevant guidelines and regulations.

## Results

### HEV samples

To evaluate the newly established molecular methods, a total of nine plasma samples and one faecal sample from patients with clinically confirmed chronic hepatitis E (CHE) were used in this study. All these CHE infections were observed in immunosuppressed patients. (Table 1). The samples were collected between October 2017 and April 2018 with viral loads ranging between 10^5^ and 10^9^ copies/ml (Table 1). Seven samples from CHE patients, including *sample 1* and *2* (Table 1), were used to test the lrPCR method and subsequently the multiplex sequencing method using 1D^2^ flow cell from ONT. Four samples (1a, 1b, 2, and 3, Table 1) collected from one CHE patient representing three time-points of the CHE infection (follow-up samples) were used to assess the amplicon-based NGS methods. In addition, the lrPCR products from *sample 1* and *2* were further sequenced with the Illumina WG method and additionally by Sanger sequencing (data not shown), that confirmed the mutations with high frequency and the most frequent and prevalent insertions.

### Long-range PCR and Illumina sequencing

**Figure 1:**
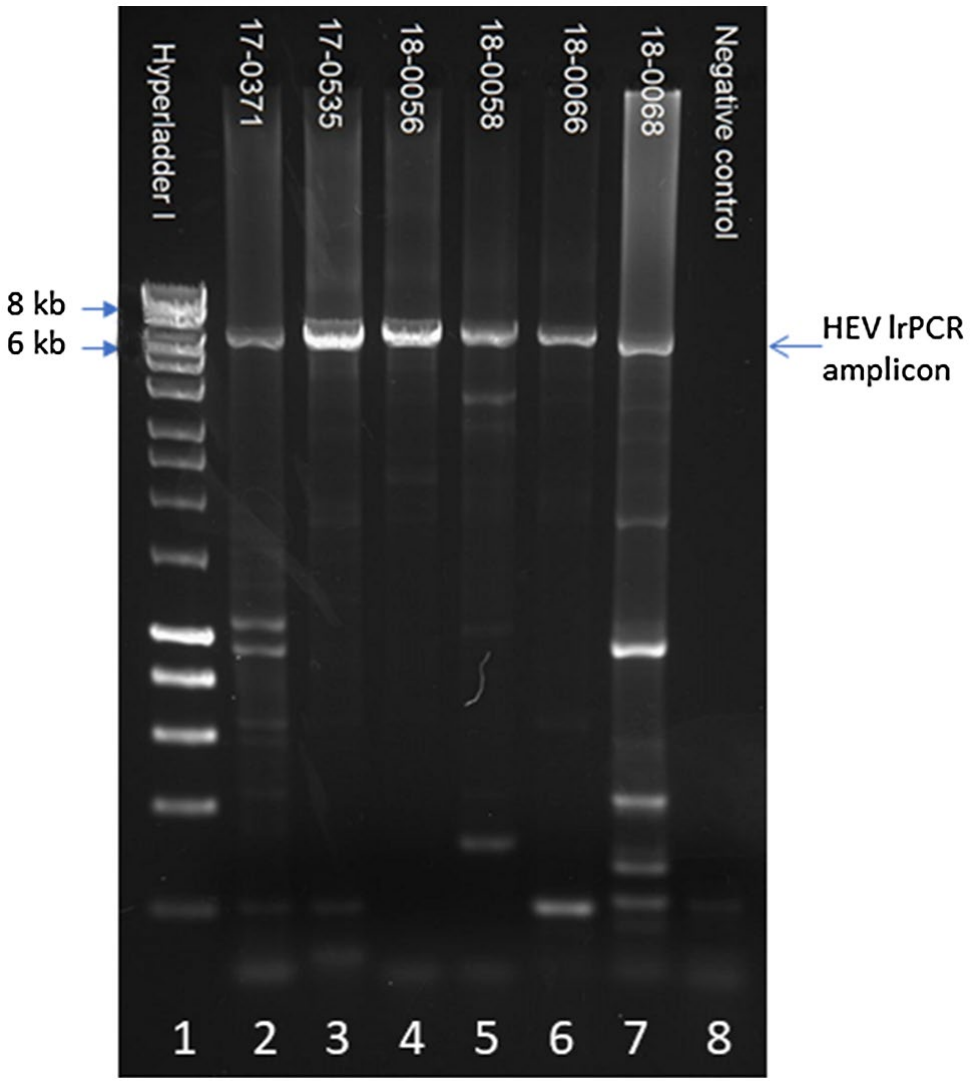
Agarose gel electrophoresis of HEV lrPCRs. Lanes 2–7 show lrPCR results of patient samples 17-0371 to 18-0068 with signals of correct size between 6 and 8 kb. Lane 1: size marker (HyperLadder™ 1 kb, Bioline)(for the creation of the gel image the software BioDocAnalyze, Version 2.67.5.0, www.biometra.com was used).

The near full-length HEV genome amplification was successful for all samples (Fig. 1; an unprocessed image of Fig. 1 is presented in the Supplementary Information). To gain a deeper insight into the diversity of viral variants, the lrPCR products from two samples (*sample 1* and *sample 2*) collected from the same patient at a six-month interval were sequenced with Illumina technology. More than two million reads were generated for each sample. After processing the raw data, 99.9% of the reads successfully mapped onto the reference with a mean coverage of approximately 60 thousand and minimum coverage of approximately 12 thousand (Supplementary Table S4). Two insertions in the HVR were detected using Illumina NGS in both samples analysed. One insertion was identified as a fragment of the human AHNAK gene, and one as a fragment from the RdRp region of the HEV genome. The exact sequences and their characteristics are presented in Tables 2 and 3.

**Table 2:** HVR insertions.

Insertion	Accession No	Sequence (5’-3’)
AHNAK^b^–WG-NGS	MW837253	TGACATAACAGGTCCAAAAGTTGATATTAATATCGAAGGCAAGTCAAAGAAATCTCGTTTTAAGCTTCCCAAATTTAATTTTTCGGGCTCTAAAGTTCAGACACTTGAAGTGGATGTCAAAGGTAAAAAACCAGAAAT
HEV–RdRp^a^—WG-NGS	MW837246	CGGTCGGCCTGGATCTTACAGGCGCCGAAGGTGTCTCTTAAGGGTTTTTGGAAGAAGCATTCTGGTGAGCCTGGTACCCTCCTTTCGACAGCATCCGCCTCCCCTGCCCCTGAGCCCGCTCAACCACCTGGCTCCGCTGGGCCAAAGACTCCTGTGCGTAAG
AHNAK^b^–Amplicon-NGS	MW837244	TGACATAACAGGTCCAAAAGTTGATATTAACATCGAAGGCAAGTCAAAGAAATTTCGTTTTAAGCTTCCCAAATTTAATTTTTCGGGCTCTAAAGTTCAGACACCTGAAGTGGATGTCAAAGGTAAAAAGCCAGATAT
RPL18^c^ (1)–Amplicon-NGS	MW837245	TCTAAGAGGTTGTTTATGAGTCGCACCAACCGGCCGCCTCTGTCCCTTTCCCGGATGATCCGGAAGATGAAGCTCCCTGGCCGGGGAAACAAGACGGCCGTGGCTGTGGGGACCATAACTGATGATGTGCGGGTTCAGGAGGTACCCAAA
RPL18^c^ (2)–Amplicon-NGS	MW837248	CTTTCCCGGATGATCCGGAAGATGAGGCTTCCTGGCCGGGAAAACAAGACGGCCGTGGCTGTGGGGACCATAACTGATGATGTGCGGGTTCAGGAGGTACCCAAA
HEV–RdRp^a^–Amplicon-NGS	MW837246	CGGTCGGCCTGGATCTTACAGGCGCCGAAGGTGTCTCTTAAGGGTTTTTGGAAGAAGCATTCTGGTGAGCCTGGTACCCTCCTTTCGACAGCATCCGCCTCCCCTGCCCCTGAGCCCGCTCAACCACCTGGCTCCGCTGGGCCAAAGACTCCTGTGCGTAAG
HEV—HVR duplication^a^– Amplicon-NGS	MW837247	GCTGGGCCAAAGACTCCCGTGCGTAAGCCGCCAACGCCACCACCCCCGCGCACCCGCCGC

^a^Using wbGER27 as reference (accession No: FJ705359.1); ^b^homo sapiens AHNAK nucleoprotein (human neuroblast differentiation-associated protein (desmoyokin), accession No.: NG_051822.1); ^c^homo sapiens ribosomal protein L18 (RPL18, accession No.: L11566.1); HVR = hypervariable region; NGS = next generation sequencing; WG-NGS = whole genome next generation sequencing.

**Table 3:** BLASTn results of the insertions.

Source of Insertion	Position in HEV^a^ (bp)	Length (bp)	BLASTn Results Position on Insertion: Position in Source (bp)	
**Whole-genome NGS**				
AHNAK^b^	2,227	138	Segment 1: 1–30:27,578–27,607 Identity with NG_051822.1: 100% Mismatches: 0	Segment 2: 27–138:27,466–27,577 Identity with NG_051822.1: 97.3% Mismatches: 3
HEV–RdRp^a^	2,360	162	Segment 1: 1–88:4,544–4,631 Identity with FJ705359.1: 95.5% Mismatches: 4	Segment 2: 88–162:2,285–2,359 Identity with FJ705359.1: 88% Mismatches: 9
**Amplicon-based NGS**				
AHNAK^b^	2,227	138	Segment 1: 1–30:27,578–27,607 Identity with NG_051822.1: 100% Mismatches: 0	Segment 2: 27–138:27,466–27,577 Identity with NG_051822.1: 98.2% Mismatches: 2
RPL18^c^, variant 1	2,273	150	4–150:160–306 Identity with L11566.1: 98% Mismatches: 3	
RPL18-2^c^, variant 2	2,246	105	1–105:202–306 Identity with L11566.1: 98.1% Mismatches: 2 deletion of HEV sequence from 2,246 to 2,275	
HEV–RdRp^a^	2,360	162	Segment 1: 1–88:4,544–4,631 Identity with FJ705359.1: 95.5% Mismatches: 4	Segment 2: 88–162:2,285–2,359 Identity with FJ705359.1: 88% Mismatches: 9
HEV–HVR^a^	2,393	60	1–60:2,333–2,392 Identity with FJ705359.1: 96.7% Mismatches: 2	

^a^Using wbGER27 as reference (accession No: FJ705359.1); ^b^homo sapiens AHNAK nucleoprotein (accession No.: NG_051822.1); ^c^homo sapiens ribosomal protein L18 (accession No.: L11566.1).

### Long-read sequencing with Oxford Nanopore technology

**Figure 2:**
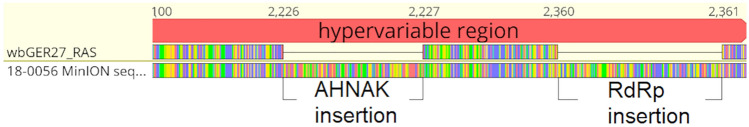
Cumulated AHNAK- and RdRp-derived Insertions in the HVR. The red bar represents the sequence annotation. Reference sequence is wbGER27_RAS (FJ705359.1) and below the sequence containing two insertions (AHNAK and RdRp) detected in sample 18-0056 (*sample 2*) using long-read sequencing. Visualization of data by Geneious version 11.1.5 (Biomatters Ltd, Auckland, NZ).

The 1D^2^ flow cell generated a mean yield of approximately 285,000 reads per sample. The number of reads per sample after size selection ranged from 11,000 to more than 68,000 reads (Supplementary Table S5). When mapped to the HEV reference the reads compared well to the length of the PCR products covering 95% of the HEV genome. Thus, near full-length sequences of six samples were generated using long-read sequencing with the MinION device. The two insertions identified by Illumina NGS were also detected when analysing the MinION reads of *sample 1* and *2*. This confirmed the coexistence of both variants at an early time-point of HEV infection. Interestingly, we were able to detect in *sample 2* five ONT reads showing both the AHNAK-derived and the RdRp-derived insertions, separated by a 132 bp HEV specific connecting sequence (Fig. 2). To validate the detection of the HVR variant with simultaneous AHNAK- and RdRp-derived insertions also in the Illumina reads, the Illumina reads from *sample* 2 were mapped against ONT reads. Thus, Illumina reads were detected that contained the 3´-end of the AHNAK insertion, the whole HEV specific connecting sequence, and the 5´end of the RdRp insertion. Moreover, approximately 0.1% of the Illumina reads covering this specific region of the HVR contained parts of both AHNAK- and RdRp-derived sequences simultaneously. This confirms the double-insertion variant showing that HEV can cumulate insertions.

### Targeted NGS

For the targeted NSG approach we used *sample 1* which was a plasma (sample 1a) and a stool sample (sample 1b) from the same patient and same time-point (Table 1). The read qualities and coverages of the HVR amplicons after mapping to the reference wbGER27_RAS (FJ705359.1) are shown in Table S6. Here, we detected further insertions in addition to the ones found by whole-genome sequencing (Tables 2, 3, and 4). The insertions are all in-frame and range between 21 and 54 amino acids in length. A more detailed analysis showed that one insertion derives from the AHNAK nucleoprotein sequence from the human genome, two were from the human ribosomal protein L18 sequences, one has its origin in the RdRp of the HEV genome, and one is a duplication of 21 amino acids of the HVR. The sequences of the two human RPL18 insertions are identical, however, one sequence lacks the first 15 amino acids and is preceded by a deletion of the HEV sequence. Notably, the truncated RPL18-derived insertion was detected only in the stool sample (Table 4). Using amplicon-based NGS sequencing a total of five insertions in the stool and three insertions in the plasma sample could be detected compared to Illumina WG or MinION sequencing where only the AHNAK and RdRp derived insertions could be identified. To confirm the high extent of variability detected in the HVR we additionally sequenced the more conserved RdRp region from the same samples using the amplicon-based NGS method. Since mutations in the RdRp region could be associated with therapy failure, a targeted deep-sequencing approach for this region is useful for mutational analysis. Read quality and coverage of the RdRp amplicons after mapping against wbGER27_RAS (FJ705359.1) are shown in Table S7. The RdRp reads showed no structural changes besides polymorphisms. The variability of sample 1a and 1b of both HVR and RdRp amplicons was compared. The number of polymorphic loci for both HVR and RdRp showed a ratio approximately twice as high for the HVR (1a: 0.37 and 1b: 0.41) compared to the RdRp (1a: 0.22 and 1b: 0.17). Furthermore, the ratio between non-synonymous SNPs and synonymous SNPs was 1.5 and 1.2 for HVR compared to 0.5 and 0.23 for the RdRp, respectively. This finding indicated that the HVR is under positive selection whereas the RdRp is under negative selection. Notably, sample 1b had a nine times higher mean coverage than 1a for the RdRp amplicons and a seven times higher mean coverage for the HVR amplicon (Table S7). In addition, all insertions described above could be detected also in the pooled HVR and RdRp amplicons. The read quality and coverage of the pooled amplicons after mapping against wbGER27_RAS (FJ705359.1) are shown in Table S7.

**Table 4:** Proportions of reads supporting insertions.

Sequencing method	Insertion	Sample	Reads containing insertion/total reads	Percentage of reads containing insertion
Whole-genome NGS	AHNAK^b^	*Sample 1*	36,170/42,760	~ 85%
		*Sample 2*	4,770/23,410	~ 20%
	HEV–RdRp^a^	*Sample 1*	1,360/42,760	~ 3%
		*Sample 2*	5,590 /23,410	~ 24%
Amplicon-based NGS	AHNAK^b^	*Sample 1*	620/15,980	~ 4%
		Sample 1b	31,840/135,350	~ 23%
	RPL18^c^, variant 1	*Sample 1*	5,210/15,980	~ 33%
		Sample 1b	47,220/135,350	~ 35%
	RPL18-2^c^, variant 2	*Sample 1*	0	0%
		Sample 1b	1,580/135,350	~ 1%
	HEV–RdRp^a^	*Sample 1*	20/15,980	< 1%
		Sample 1b	1,280/135,350	~ 1%
	HEV–HVR^a^	*Sample 1*	0	0%
		Sample 1b	814/135,350	< 1%

^a^Using wbGER27 as reference (accession No: FJ705359.1); ^b^homo sapiens AHNAK nucleoprotein (accession No.: NG_051822.1); ^c^homo sapiens ribosomal protein L18 (accession No.: L11566.1).

### Dynamics of SNPs

The RdRp region of five samples (1a, 1b, 2, 3, and 4, Table 1) from one patient under ribavirin therapy collected within six months was analysed. The frequency of mutations in the RdRp region found in these samples that are associated with therapy failure or higher replication, namely K1383N, V1479I, Y1587F, and G1634R are shown in Table 5. Notably, between the second and third time-points all specified mutations increased, especially Y1587F and K1383N. The mutations Y1587F and K1383N increased from values lower than the cut-off to approximately 90% and 98% of reads at time-point three. At time-point four, the percentages of G1634R and V1479I increased further, whereas Y1587F and K1383N maintained consistent. When determining the frequencies of the RdRp mutations for each insertion group using the ONT reads there was no difference in the distribution of these mutations compared to the initial analysis where all sequences were assessed together. Therefore, there is most likely no correlation between insertion in the HVR and mutations in the RdRp.

**Table 5:** RdRp mutations in percent.

Sample no	Time-point	Weeks after 1st Time-point	G1634R	Y1587F	V1479I	K1383N
1a (*sample 1*)	1—blood	0	1.1	< cut-off	< cut-off	< cut-off
1b	1—stool	0	1.1	< cut-off	< cut-off	< cut-off
2	2	6	< cut-off	< cut-off	< cut-off	< cut-off
3	3	12	6.5	90	2	98
4 (*sample 2*)	4	25	50	85	8.5	100

## Discussion

To gain insights into the mechanisms leading to pathophysiology, persistence, and adaptation of HEV in their hosts and to improve clinical outcome as well as management of HEV infections, the need for robust methods that facilitate the use of cutting-edge technologies is obvious. Amplifying the whole-genome by lrPCR allows sequencing of viral strains as single molecules using third-generation sequencing. Whole-genome sequencing is closer to physiology compared to smaller amplicons and fragments while it reduces the number of PCRs needed, the preparation costs, and saves time. Using lrPCR products for NGS we obtained a high coverage throughout the whole HEV genome which makes this method suitable for intra-populational analysis. Increasing the amount of target cDNA and validating mutations using data from follow-up samples a cut-off value for variant detection as low as 0.5% can be applied. Exemplarily for this study, proportions of known minority variants, such as G1634R, were compared in *sample 1* (plasma sample) and sample 1b (stool sample) which were collected at the same time-point and sequenced using different approaches. Consequently, we were able to validate and compare the number of mutations in the HEV genome by different methods despite the low cut-off value of 0.5%. However, regarding the detection of long insertions, single-molecule sequencing by ONT was superior to whole genome NGS with the Illumina technology as the long-reads of the ONT captured a viral variant containing AHNAK- and RdRp-derived insertions which cumulated on the same strand. This is the first report of such a mixed HVR viral variant. Furthermore, a correlation between mutations in the RdRp and recombination events in the HVR could be excluded. This indicated that mutation selection and recombination events are probably unlinked mechanisms that drive viral diversity and adaptation through different molecular mechanisms highlighting the advantage of long-read sequencing technologies. Nevertheless, it is worth mentioning that third-generation sequencing has a higher error rate than NGS, which makes this method less suitable for the detection of polymorphisms^27^.

With regard to the detection of insertions, the amplicon-based sequencing proved to be the method of choice. The amplicon-based sequencing showed the best performance by detecting the most insertions if compared to whole-genome sequencing; however, due to the 2 × 300 bp reads and thus smaller amplicon size, this method failed to detect the HEV variant with two insertions that occur on the same strand with a total length of 432 bp which could be detected only by third-generation sequencing. Both NGS methods presented allowed for a very detailed analysis; however, only the AHNAK and RdRp derived insertions were detected when sequencing was carried out using lrPCR. This may be due to the cDNA synthesis which was performed using Oligo d(T)s for the lrPCR whereas for the HVR amplicon random hexamers were used which tend to achieve higher cDNA yield^24^. Sampling bias, different cDNA synthesis methods, and a potential strand selection of the PCR primers could explain the differences in the proportion of the insertions when comparing the whole-genome NGS and the amplicon-based NGS datasets. Therefore, the frequencies given for each insertion were listed for completeness and should be interpreted accordingly. While standard sample preparation for NGS includes tagmentation which can lead to sequencing of host DNA and thus artifacts that could be misinterpreted as insertions, the amplicon-based sequencing method was performed without tagmentation. This approach aimed to generate PCR products and subsequently reads that detected both HEV specific sequence of the HVR region with possible insertion. The PCR products were generated with target-specific primers designed to contain the flow cell adapters. In addition, the generated reads were clustered and consensus sequences of highly similar reads were generated. Therefore, to exclude contamination by host DNA which may also have been sequenced, only insertions were validated where the reads simultaneously contained the insertion and HEV-specific sequences. Moreover, the AHNAK insertion was confirmed by long-read sequencing, indicating that human sequences were integrated into the viral genome. Insertions originating in the human genome have previously been described in HEV and some of these HEV variants have been shown to efficiently replicate in the human hepatocyte cell line HepG2/C3A^7,28,29^. Insertions in the HEV genome and particularly in the HVR region have already been detected in samples of CHE individuals and additionally, although very rarely, in samples from individuals with acute HEV infection, suggesting a potential role of HVR diversity in chronification^11,12,30^. However, the impact of these recombination events in the HVR region on the chronic course of infection, the stability of viral genome and viral replication remains unclear. Using the amplicon-based method five different insertions in the HVR could be detected in one sample. Two insertions derived from the human RPL18 gene showing identical sequences; however, one of these lacked 15 amino acids from the RPL18 insertion and nine amino acids from the HEV sequences that precedes the insertion. A possible explanation for this truncated RPL18 insertion is that HEV highjacked the RPL18 gene after which a deletion took place. This finding is in accordance with a recent report where 114 HEV strains have been sequenced using PacBio platforms and insertions from human genes or duplications of the HEV genome were detected in seven patients. However, in one sample six nucleotides truncated human gene fragment RNA18SP5 was detected using single-molecule sequencing^11^. Besides the possibility of sequencing error, Lhomme et al. also discussed the possibility of different biological variants that have been detected separately by different sequencing methods^11^. As noted above, some recombination variants are likely missed using different sequencing approaches due to their low quantity in the viral population. However, using the amplicon-based ultra-deep sequencing method of the HVR, we were able to detect not just one but five different insertions in the same sample. It is therefore evident that amplicon-based ultra-deep sequencing is best suited for the screening of the HVR of many samples allowing multiplexing, high accuracy, and ultra-deep sequencing.

In contrast to the HVR, the RdRp region is highly conserved and could be used to validate HVR sequencing. The RdRp region is clinically relevant due to the variants that can occur by the selection of mutations associated with higher replication competence and potential therapy failure^10,17,18^. The HVRs in samples 1a (blood) and 1b (stool) were under positive selection which suggests that amino acid changes in this region may play an important role for the virus replication and fitness. Positive selection in the HVR has been described previously and it seems to be a characteristic of the zoonotic HEV genotypes HEV-3 and HEV-4^6^. Considering the quality and validity of our data, a cut-off of 0.5% for variant detection was implemented focusing on the dynamics of the mutations. In both, sample 1a (plasma) and 1b (stool), the mutation G1634R was detected at 1.1% confirming the robustness of the method. Notably, the G1634R mutation was a minority mutation that was detectable already at the first time-point at a low level and was being selected in the course of infection. Using the amplicon sequencing method for sample analyses of other patients the mutations K1383N and G1634R could be detected in low frequencies even before the initiation of antiviral treatment^31^ or in increased frequencies after sofosbuvir and ribavirin combination therapy failure^32^. The RdRp mutations presented in Table 5 are known to be selected during ribavirin therapy as previously reported^10,18^. Interestingly, in the samples analysed here, Y1587F and K1383N mutations appeared within six weeks between the second and third time-point and increased subsequently to 85 and 100%, respectively. Thus, the amplicon-based deep sequencing method has proven useful in capturing the dynamics of mutations in patients with CHE. Notably, this method allows pooling of amplicons from RdRp and HVR amplification maximising the data gained and reducing the costs.

Nevertheless, the presented methods have their limitations. One limitation of the lrPCR is the lower efficiency compared to shorter PCR amplicons. Samples with low viral load might be difficult or even impossible to amplify. Further limitations of the lrPCR were determined by the incomplete coverage of the coding region and by the low number of samples used for validation and testing. A further limitation was that the HEV subtype of all samples analysed here was HEV-3c, the most prevalent subtype in Germany. However, we expect that the here described approaches will be applicable also for other subtypes; although, marginal adaptation might be necessary. Regarding sequencing, limitations of the presented methods are more specific to the sequencing technologies used. Illumina sequencing requires fragmentation of the PCR products if these products exceed the length of two paired end reads while some insertions can be missed. On the other hand, if amplicon NGS is possible designing the right amplicon size can be challenging. For instance, we saw a drop of coverage in the middle-sequences of all RdRp amplicons due to the weak overlap of the paired end reads. Amplicons should be therefore of optimal length to present an advantage over, e.g., the tagmentation method. Long-read sequencing technologies, such as ONT, have relatively high error rates which is detrimental for variant analysis. Thus, we overcame this limitation by performing simultaneous sequencing with both Illumina and ONT technologies. Furthermore, we have not undertaken any studies to show the effect of insertions on HEV replication and genome stability. Underlying mechanisms relevant to viral gene duplication of human gene recombination with the HEV genome is not clear. This may be considered as limitation of our study. In this study, we focused only on identifying and validating insertions in the HVR region to gain more detailed insights into the complexity and variation of HVR insertions by using advanced sequencing techniques.

In summary, we have shown here that lrPCR is an elegant method that facilitates whole-genome sequencing with the Illumina and ONT technologies; however, the amplicon-based deep-sequencing of the HVR and RdRp region is the method of choice in screening HEV samples for insertions or mutations. Amplicon-based deep-sequencing has the accuracy of the Illumina technology, allows multiplexing, and reduces sequencing costs. This approach should be considered especially in CHE patients where sustained virologic response cannot be achieved facing an elevated risk for the selection of viral variants with possible increased pathogenicity.

## Supplementary Information

Supplementary Information.

## Data Availability

All data needed to evaluate the conclusions in the paper are present in the manuscript and/or the Supplementary Materials. Further data will be made available to interested researchers by reasonable request to the first and/or the corresponding author.

## References

[1] Wedemeyer H, Pischke S, Manns MP (2012). Pathogenesis and treatment of hepatitis e virus infection. Gastroenterology.

[2] Kamar N, Bendall R, Legrand-Abravanel F, Xia NS, Ijaz S, Izopet J, Dalton HR, Hepatitis E (2012). Lancet.

[3] Dalton HR, Stableforth W, Thurairajah P, Hazeldine S, Remnarace R, Usama W, Farrington L, Hamad N, Sieberhagen C, Ellis V, Mitchell J, Hussaini SH, Banks M, Ijaz S, Bendall RP (2008). Autochthonous hepatitis E in Southwest England: natural history, complications and seasonal variation, and hepatitis E virus IgG seroprevalence in blood donors, the elderly and patients with chronic liver disease. Eur J Gastroenterol Hepatol.

[4] Said B, Usdin M, Warburton F, Ijaz S, Tedder RS, Morgan D (2017). Pork products associated with human infection caused by an emerging phylotype of hepatitis E virus in England and Wales. Epidemiol Infect.

[5] Spahr C, Knauf-Witzens T, Vahlenkamp T, Ulrich RG, Johne R (2018). Hepatitis E virus and related viruses in wild, domestic and zoo animals: a review. Zoonoses Public Health.

[6] Purdy MA, Khudyakov YE (2010). Evolutionary history and population dynamics of hepatitis E virus. PLoS ONE.

[7] Shukla P, Nguyen HT, Torian U, Engle RE, Faulk K, Dalton HR, Bendall RP, Keane FE, Purcell RH, Emerson SU (2011). Cross-species infections of cultured cells by hepatitis E virus and discovery of an infectious virus-host recombinant. Proc. Natl. Acad. Sci. U. S .A.

[8] Wang H, Zhang W, Ni B, Shen H, Song Y, Wang X, Shao S, Hua X, Cui L (2010). Recombination analysis reveals a double recombination event in hepatitis E virus. Virol J..

[9] Smith, D. B., Simmonds, P., Jameel, S., Emerson, S. U., Harrison, T. J., Meng, X. J., Okamoto, H., Van der Poel, W. H., Purdy, M. A. & Group, I. C. o. T. o. V. H. S. Consensus proposals for classification of the family Hepeviridae. *J Gen Virol* **95**, 2223–2232. 10.1099/vir.0.068429-0 (2014). 10.1099/vir.0.068429-0 PMC4165930 24989172

[10] Debing Y, Ramière C, Dallmeier K, Piorkowski G, Trabaud MA, Lebossé F, Scholtès C, Roche M, Legras-Lachuer C, de Lamballerie X, André P, Neyts J (2016). Hepatitis E virus mutations associated with ribavirin treatment failure result in altered viral fitness and ribavirin sensitivity. J Hepatol.

[11] Lhomme S, Nicot F, Jeanne N, Dimeglio C, Roulet A, Lefebvre C, Carcenac R, Manno M, Dubois M, Peron JM, Alric L, Kamar N, Abravanel F, Izopet J (2020). insertions and duplications in the polyproline region of the hepatitis E virus. Front. Microbiol..

[12] Lhomme S, Abravanel F, Dubois M, Sandres-Saune K, Mansuy JM, Rostaing L, Kamar N, Izopet J (2014). Characterization of the polyproline region of the hepatitis E virus in immunocompromised patients. J. Virol..

[13] Kenney SP, Meng XJ (2015). The lysine residues within the human ribosomal protein S17 sequence naturally inserted into the viral nonstructural protein of a unique strain of hepatitis E virus are important for enhanced virus replication. J. Virol..

[14] Kenney SP, Meng XJ (2015). Identification and fine mapping of nuclear and nucleolar localization signals within the human ribosomal protein S17. PLoS ONE.

[15] Johne R, Reetz J, Ulrich RG, Machnowska P, Sachsenröder J, Nickel P, Hofmann J (2014). An ORF1-rearranged hepatitis E virus derived from a chronically infected patient efficiently replicates in cell culture. J. Viral Hepat..

[16] Lhomme S, Garrouste C, Kamar N, Saune K, Abravanel F, Mansuy JM, Dubois M, Rostaing L, Izopet J (2014). Influence of polyproline region and macro domain genetic heterogeneity on HEV persistence in immunocompromised patients. J Infect Dis.

[17] Debing, Y., Gisa, A., Dallmeier, K., Pischke, S., Bremer, B., Manns, M., Wedemeyer, H., Suneetha, P. V. & Neyts, J. A mutation in the hepatitis E virus RNA polymerase promotes its replication and associates with ribavirin treatment failure in organ transplant recipients. *Gastroenterology* **147**, 1008–1011.e1007; quiz e1015–1006. 10.1053/j.gastro.2014.08.040 (2014). 10.1053/j.gastro.2014.08.040 25181691

[18] Todt D, Gisa A, Radonic A, Nitsche A, Behrendt P, Suneetha PV, Pischke S, Bremer B, Brown RJ, Manns MP, Cornberg M, Bock CT, Steinmann E, Wedemeyer H (2016). In vivo evidence for ribavirin-induced mutagenesis of the hepatitis E virus genome. Gut.

[19] Lauring AS, Andino R (2010). Quasispecies theory and the behavior of RNA viruses. PLoS Pathog.

[20] Mohamed S, Penaranda G, Gonzalez D, Camus C, Khiri H, Boulmé R, Sayada C, Philibert P, Olive D, Halfon P (2014). Comparison of ultra-deep versus Sanger sequencing detection of minority mutations on the HIV-1 drug resistance interpretations after virological failure. AIDS.

[21] EASL Clinical Practice Guidelines on hepatitis E virus infection. *J Hepatol* **68**, 1256–1271. 10.1016/j.jhep.2018.03.005 (2018). 10.1016/j.jhep.2018.03.005 29609832

[22] Wang, B., Harms, D., Papp, C. P., Niendorf, S., Jacobsen, S., Lütgehetmann, M., Pischke, S., Wedermeyer, H., Hofmann, J. & Bock, C. T. Comprehensive Molecular Approach for Characterization of Hepatitis E Virus Genotype 3 Variants. *J Clin Microbiol* **56**. doi:10.1128/JCM.01686-17 (2018). 10.1128/JCM.01686-17 PMC5925713 29514938

[23] Jothikumar N, Cromeans TL, Robertson BH, Meng XJ, Hill VR (2006). A broadly reactive one-step real-time RT-PCR assay for rapid and sensitive detection of hepatitis E virus. J Virol Methods.

[24] Zucha D, Androvic P, Kubista M, Valihrach L (2019). Performance Comparison of Reverse Transcriptases for Single-Cell Studies. Clin Chem..

[25] Zhao C, Liu F, Pyle AM (2018). An ultraprocessive, accurate reverse transcriptase encoded by a metazoan group II intron. RNA.

[26] Edgar, R. C. UNOISE2: improved error-correction for Illumina 16S and ITS amplicon sequencing. *bioRxiv*, 081257. 10.1101/081257 (2016).

[27] Laver T, Harrison J, O'Neill PA, Moore K, Farbos A, Paszkiewicz K, Studholme DJ (2015). Assessing the performance of the Oxford Nanopore Technologies MinION. Biomol Detect Quantif.

[28] Shukla P, Nguyen HT, Faulk K, Mather K, Torian U, Engle RE, Emerson SU (2012). Adaptation of a genotype 3 hepatitis E virus to efficient growth in cell culture depends on an inserted human gene segment acquired by recombination. J Virol.

[29] Nguyen HT, Torian U, Faulk K, Mather K, Engle RE, Thompson E, Bonkovsky HL, Emerson SU (2012). A naturally occurring human/hepatitis E recombinant virus predominates in serum but not in faeces of a chronic hepatitis E patient and has a growth advantage in cell culture. J Gen Virol.

[30] Munoz-Chimeno, M., Cenalmor, A., Garcia-Lugo, M. A., Hernandez, M., Rodriguez-Lazaro, D. & Avellon, A. Proline-rich hypervariable region of hepatitis e virus: Arranging the disorder. *Microorganisms* **8**. 10.3390/microorganisms8091417 (2020). 10.3390/microorganisms8091417 PMC7564002 32942608

[31] Gerhardt, F., Maier, M., Liebert, U. G., Platzbecker, U., Wang, S. Y., Papp, C. P., Bock, C. T., Berg, T. & van Bömmel, F. Early Detection of Hepatitis E Virus Ribavirin Resistance Using Next-Generation Sequencing. *Antimicrob Agents Chemother* **64**. doi:10.1128/AAC.01525-19 (2019). 10.1128/AAC.01525-19 PMC7187607 31685465

[32] Schulz M, Papp CP, Bock CT, Hofmann J, Gerlach UA, Maurer MM, Eurich D, Mueller T (2019). Combination therapy of sofosbuvir and ribavirin fails to clear chronic hepatitis E infection in a multivisceral transplanted patient. J Hepatol.

