# Supplementary material for: Advanced sequencing approaches detected insertions of viral and human origin in the viral genome of chronic hepatitis E virus patients
Source: Sci Rep. 2022 Feb 2;12:1720. doi: 10.1038/s41598-022-05706-w (PMC8811047; doi:10.1038/s41598-022-05706-w)
Supplement: Supplementary file 1 — Supplementary Information. [file 41598_2022_5706_MOESM1_ESM.docx]

**Supplementary information**

**Advanced sequencing approaches detected insertions of viral and human origin in the viral genome of chronic hepatitis E virus patients**

C.-Patrick Papp, Paula Biedermann, Dominik Harms, Bo Wang, Marianne Kebelmann, Mira Choi, Johannes Helmuth, Victor M. Corman, Andrea Thürmer, Britta Altmann, Patrycja Klink, Jörg Hofmann, C.-Thomas Bock

**Table S1.** Protocols for genom-wide and long-range PCRs (lrPCR)

| **Method** | **Denaturation** | **Amplification** | | **Final elongation** |
| --- | --- | --- | --- | --- |
|  |  | **Cycles** |  |  |
| cDNA synthesis test | 5 min / 95°C | 10x | 30 sec / 94°C | 1 min / 72°C |
|  |  |  | 30 sec / 60°C  -1℃ per cycle |  |
|  |  |  | 45 sec / 72°C |  |
|  |  | 30x | 30 sec / 94°C |  |
|  |  |  | 30 sec / 52°C |  |
|  |  |  | 45 sec / 72°C |  |
| long-range PCR | 5 min / 95°C | 35x | 20 sec / 98°C | 5 min / 74°C |
|  |  |  | 8 min / 74°C |  |
| HVR 1^st^ PCR | 5 min / 95°C | 35x | 20 sec / 98°C | 1 min / 68°C |
|  |  |  | 30 sec / 68°C |  |
| HVR nested PCR | 5 min / 95°C | 35x | 20 sec / 98°C | 1 min / 72°C |
|  |  |  | 15 sec / 62°C |  |
|  |  |  | 1 min / 72°C |  |
| RdRp 1st PCR | 2 min / 94°C | 35x | 30 sec / 94°C | 1 min / 72°C |
|  |  |  | 30 sec / 48°C |  |
|  |  |  | 2.5 min / 72°C |  |
| RdRp nested PCR | 2 min / 94°C | 35x | 30 sec / 94°C | 1 min / 68°C |
|  |  |  | 1.5 min / 68°C |  |

**Table S2.** Primers

| **Application** | **Sequence (5’-3’)** | **Direction** | **Position^a^** |
| --- | --- | --- | --- |
| cDNA synthesis test | CTGGCATYACTACTGCYATYGAGC | Forward | 54 |
| cDNA synthesis test | CCRTCRAARCARTADGTRCGRTC | Reverse | 471 |
| cDNA synthesis test | ACYTGGTCHACATCTGGYTTYTC | Forward | 2,135 |
| cDNA synthesis test | TACACCTTRGCSCCRTCRGGRTA | Reverse | 2,428 |
| cDNA synthesis test | GAGGCYATGGTSGAGAARG | Forward | 4,112 |
| cDNA synthesis test | GCCATGTTCCAGACRGTRTTCC | Reverse | 4,650 |
| cDNA synthesis test | CCGACAGAATTGATTTCGTCGG | Forward | 6,321 |
| cDNA synthesis test | GTCTTGGARTACTGCTGR | Reverse | 6,776 |
| lrPCR | AGGCCCAYCAGTTYATTAAGGCTCCTGGCATYACT | Forward | 31 |
| lrPCR | CACACCCCTGCAAACCAAGRGCGCGRCACTCCGG | Reverse | 7,086 |
| Hemi-nested lrPCR | AGGCCCAYCAGTTYATTAAGGCTCCTGGCATYACT | Forward | 31 |
| Hemi-nested lrPCR | CGGCACTCAGGGCAGAAATCATCRAAAGTRTGGG | Reverse | 7,063 |
| HVR 1^st^ PCR | GAGCTCACYCCTGCTGGYYT | Forward | 1,856 |
| HVR 1^st^ PCR | GTCTCTCGRTAYGCYGCCTC | Reverse | 2,676 |
| HVR-amplicon1^b^ | TCGTCGGCAGCGTCAGATGTGTATAAGAGACAGGGACATGGTCAACATCTGGT | Forward | 2,133 |
|  |  |  |  |
| HVR-amplicon1^b^ | GTCTCGTGGGCTCGGAGATGTGTATAAGAGACAGGCCCCATCCGGATAGGTG | Reverse | 2,418 |
| HVR-amplicon2^b^ | TCGTCGGCAGCGTCAGATGTGTATAAGAGACAGGTACACCCGKACATGGTCR | Forward | 2,125 |
| HVR-amplicon2^b^ | GTCTCGTGGGCTCGGAGATGTGTATAAGAGACAGCCGGGRTTCGAYGCRTTRA | Reverse | 2,484 |
| RdRp 1^st^ PCR | ATAGACTTYGAKCAYGCCGG | Forward | 3,236 |
| RdRp 1^st^ PCR | AACAGCAACARAAYAGCCCT | Reverse | 5,200 |
| 1^st^ RdRp amplicon^b^ | TCGTCGGCAGCGTCAGATGTGTATAAGAGACAGGGCTTGTTACGTGAGGTYGGTA | Forward | 3,617 |
| 1^st^ RdRp amplicon^b^ | GTCTCGTGGGCTCGGAGATGTGTATAAGAGACAGGATTGCARAGGTCGAGCTC | Reverse | 4,172 |
| 2^nd^ RdRp amplicon^b^ | TCGTCGGCAGCGTCAGATGTGTATAAGAGACAGATGGTRGAKAARGGTCAGGAYG | Forward | 4,118 |
| 2^nd^ RdRp amplicon^b^ | GTCTCGTGGGCTCGGAGATGTGTATAAGAGACAGCACGRAAYTCATAGCARTGTGC | Reverse | 4,674 |
| 3^rd^ RdRp amplicon^b^ | TCGTCGGCAGCGTCAGATGTGTATAAGAGACAGCACCCTYCTYTGGAAYACYG | Forward | 4,618 |
| 3^rd^ RdRp amplicon^b^ | GTCTCGTGGGCTCGGAGATGTGTATAAGAGACAGTGGYGATCCCATGGGCRATG | Reverse | 5,172 |

^a^wbGER27_RAS (FJ705359.1); ^b^Illumina flow cell overhang adapters followed by target specific sequence; R stands for G or A; Y stands for C or T; S stands for G or C; W stands for A or T; M stands for A or C; K stands for G or T; H stands for A, C, or T; and N stands for A, T, C, or G.

**Table S3.** NCBI GenBank Accession Numbers

| **Sample No.** | **GenBank Accession No.** | **Sequencing method** | **Insertion** |
| --- | --- | --- | --- |
| 1a – *Sample 1* | MW837253 | Near full-length NGS | AHNAK^b^ |
| 1a – *Sample 1* | MW837250 | Amplicon RdRp |  |
| 1b | MW837243 | Amplicon HVR |  |
| 1b | MW837244 | Amplicon HVR | AHNAK^b^ |
| 1b | MW837245 | Amplicon HVR | RPL18^c^ |
| 1b | MW837246 | Amplicon HVR | RdRp^a^ |
| 1b | MW837247 | Amplicon HVR | HVR duplication^a^ |
| 1b | MW837248 | Amplicon HVR | RPL18^c^ |
| 1b | MW837249 | Amplicon RdRp |  |
| 2 | MW837251 | Amplicon RdRp |  |
| 3 | MW837252 | Amplicon RdRp |  |
| 4 – *Sample 2* | MW837254 | Near full-length NGS |  |
| 4 – *Sample 2* | MW837255 | Near full-length ONT | AHNAK^b^ and RdRp^a^ |

^a^ reference wbGER27 (HEV): AccessionNo: FJ705359.1

^b^Homo sapiens AHNAK nucleoprotein; AccessionNo.: NG_051822.1

^c^Homo sapiens ribosomal protein L18; AccessionNo.: [L11566.1](https://www.ncbi.nlm.nih.gov/nucleotide/L11566.1?report=genbank&log$=nuclalign&blast_rank=15&RID=ZSEVSBPG016)

**Table S4**. Illumina HiSeq reads and coverage for sample 1 and 2

| **Sample No.** | **1a** | **4** |
| --- | --- | --- |
| **Sample name** | 17-0421 – *sample 1* | 18-0056 – *sample 2* |
| **Raw paired reads** | 2,201,956 | 2,430,080 |
| **Reads after trimming** | 2,092,890 | 2,330,356 |
| **Percentage of trimmed reads that mapped onto reference (FJ705359.1)** | 99.9% | 99.9% |
| **Percentage of the reference sequence that was covered by reads** | 96.2% | 96.2% |
| **Mean length of reads (bp)** | 206 | 203 |
| **Mean coverage** | 61,379 | 64,996 |
| **Maximum coverage** | 96,508 | 92,174 |
| **Minimum coverage** | 11,858 | 9,792 |

**Table S5**. Number of demultiplexed ONT reads per sample

| **Sample No.** | **Sample name** | **Raw reads after demultiplexing** | **Size selected (6.5-8kb) reads** |
| --- | --- | --- | --- |
| **1a** | 17-0421 – sample 1 | Sequenced separately | 2,504 |
| **4** | 18-0056 – sample 2 | 135,190 | 68,598 |
| **5** | 17-0371 | 348,769 | 11,519 |
| **6** | 17-0535 | 106,353 | 41,790 |
| **7** | 18-0058 | 263,661 | 15,892 |
| **8** | 18-0066 | 194,072 | 30,242 |
| **9** | 18-0068 | 668,968 | 15,247 |
| **Unsorted reads** |  | 271,193 | 23,396 |

**Table S6**. Illumina reads and coverage of the HVR amplicon

| **Sample No.** | **1a** | **1b** |
| --- | --- | --- |
| **Sample name** | *Sample 1* | 17-0420 |
| **Raw paired reads** | 82,358 | 377,248 |
| **Reads after trimming** | 67,648 | 337,156 |
| **Percentage of trimmed reads that mapped onto reference (FJ705359.1)** | 74.6% | 98.9% |
| **Mean coverage** | 17,562 | 128,737 |
| **Maximum coverage** | 19,695 | 158,043 |
| **Minimum coverage** | 16,035 | 102,838 |

**Table S7**. Illumina reads and coverage of the RdRp amplicons

| **Sample No.** | **1a** | **1b** | **1b** | | **2** | **3** |
| --- | --- | --- | --- | --- | --- | --- |
| **Sample name** | ***Sample 1*** | **17-0420** | **17-0420 pool** | | **17-0534** | **18-0002** |
|  |  |  | **RdRp** | **HVR** |  |  |
| **Raw paired reads** | 22,772 | 161,926 | 264,364 | | 122,550 | 67,128 |
| **Reads after trimming** | 20,929 | 148,190 | 254,371 | | 103,410 | 61,273 |
| **Percentage of trimmed reads that mapped onto reference (FJ705359.1)** | 99.9% | 98.4% | 99.6% | | 99.9% | 99.9% |
| **Mean coverage** | 2,479 | 22,102 | 30,205 | 15,663 | 12,388 | 7,099 |
| **Maximum coverage** | 6,313 | 56,678 | 76,510 | 20,421 | 30,631 | 18,375 |
| **Minimum coverage** | 13 | 110 | 115 | 11,018 | 74 | 52 |


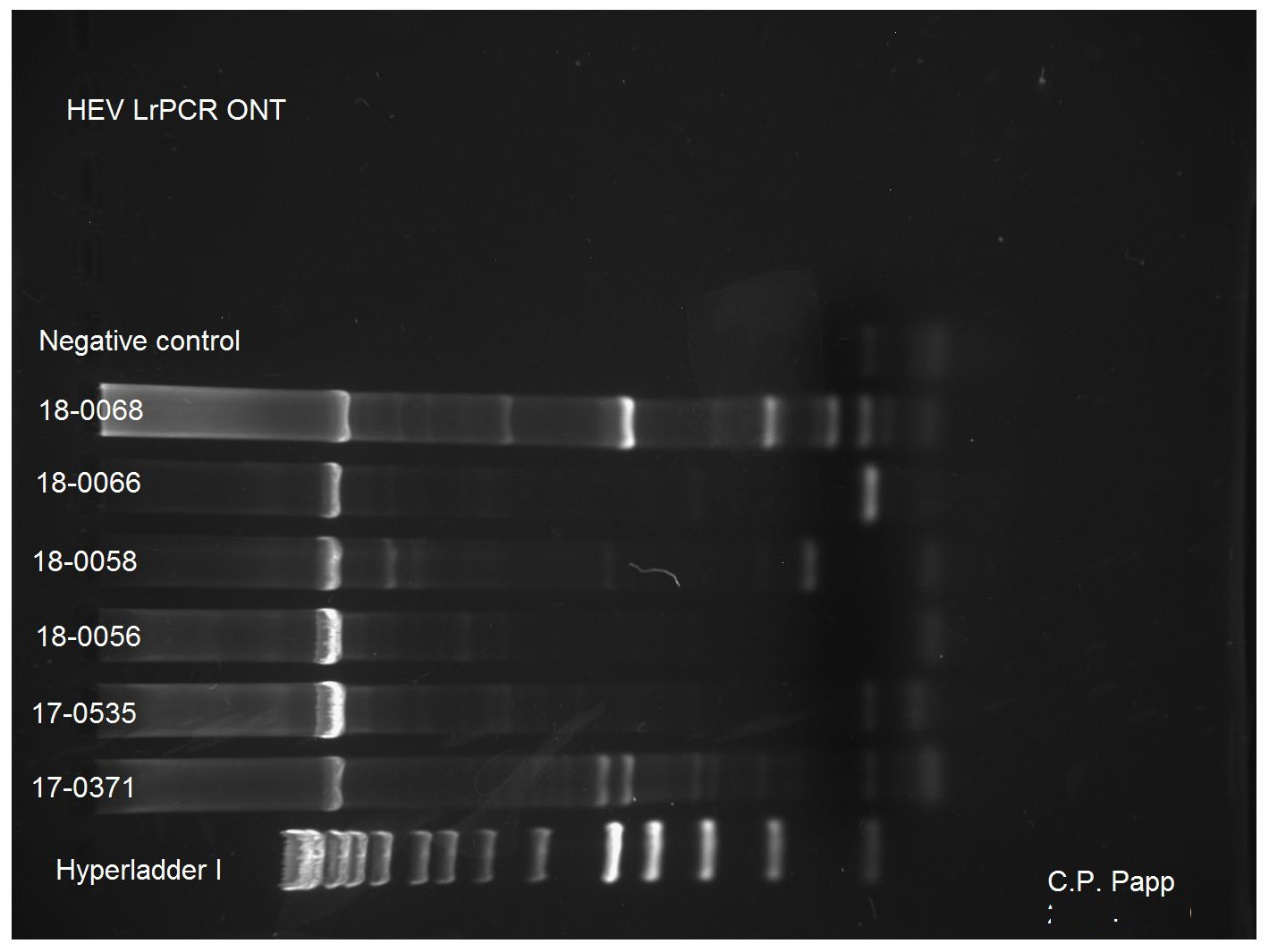
**Supplementary Figure 1.** Full-length agarose gel of HEV lrPCRs. Uncropped and unprocessed version of Fig. 1 in the main text. For the creation of the gel image the software BioDocAnalyze, Version 2.67.5.0, www.biometra.com was used.
